# Tetraspanin 6: A novel regulator of hippocampal synaptic transmission and long term plasticity

**DOI:** 10.1371/journal.pone.0171968

**Published:** 2017-02-16

**Authors:** Isabel H. Salas, Zsuzsanna Callaerts-Vegh, Amaia M. Arranz, Francesc X. Guix, Rudi D’Hooge, José A. Esteban, Bart De Strooper, Carlos G. Dotti

**Affiliations:** 1 VIB Center for Biology of Disease – VIB, Leuven, Belgium; 2 Center of Human Genetics and Leuven Institute for Neurodegenerative Diseases (LIND), KU Leuven, Leuven, Gasthuisberg O&N4, Belgium; 3 Laboratory of Biological Psychology, KU Leuven, Leuven, Belgium; 4 Centro de Biologıa Molecular ‘Severo Ochoa’ (CSIC/UAM), Madrid, Spain; Centre National de la Recherche Scientifique, FRANCE

## Abstract

Tetraspanins (Tspan) are transmembrane proteins with important scaffold and signalling functions. Deletions of *Tetraspanin 6* (*Tspan6)* gene, a member of the tetraspanin family, have been reported in patients with Epilepsy Female-restricted with Mental Retardation (EFMR). Interestingly, mutations in *Tspan7*, highly homologous to *Tspan6*, are associated with X-linked intellectual disability, suggesting that these two proteins are important for cognition. Considering recent evidences showing that Tspan7 plays a key role in synapse development and AMPAR trafficking, we initiated the study of Tspan6 in synaptic function using a *Tspan6* knock out mouse model. Here we report that hippocampal field recordings from *Tspan6* knock out mice show an enhanced basal synaptic transmission and impaired long term potentiation (LTP). A normal paired-pulse facilitation response suggests that Tspan6 affects the properties of the postsynaptic rather than the presynaptic terminal. However, no changes in spine morphology or postsynaptic markers could be detected in *Tspan6* KO mice compared with wild types. In addition, *Tspan6* KO mice show normal locomotor behaviour and no defects in hippocampus-dependent memory tests.

## Introduction

Epilepsy Female-restricted with Mental Retardation (EFMR) is a neurological disorder characterized by its remarkable X-linked inheritance: only females carrying heterozygous mutations in one of their X chromosomal alleles have the disorder, while hemizygous males are unaffected [1]. Patients show epileptic seizures that vary in type and severity and are usually accompanied by behavioral problems including autistic, obsessive and aggressive features [2]. *PCDH19* gene, encoding protocadherin 19, is the best candidate responsible for the disease with several mutations and deletions associated with the disorder [3]. However, there are still many female patients with the clinical alterations and no mutations in *PCDH19*, suggesting that other genes can also be involved in the pathology [4].

Two of the EFMR patients screened presented wide genomic deletions that spanned for *PCDH19* and six other neighboring genes: *TNMD*, *SRPX2*, *TSPAN6*, *STYL4*, *CSTF2*, *NOX1* [3,5]. Given the necessity for the identification of new genes associated with the disorder, we analyzed the deleted genes as potential candidates. One of these genes was *Tetraspanin 6* (*Tspan6*), a member of a large family of evolutionary conserved transmembrane proteins called tetraspanins. In mammals there are 33 members expressed in different cell types with diverse biological functions including cell migration and morphology, motility, cell fusion and signaling [6].

Tetraspanins are mainly expressed at the plasma membrane, which is a dynamic cellular organelle with a highly specific lipid and protein organization. It is generally accepted that the plasma membrane is compartmentalized in functional microdomains that are essential for signal transduction, membrane trafficking and cell to cell communication [7]. Particularly, synaptic membranes are characterized by a thickening of the postsynaptic membrane forming an electron-dense domain known as postsynaptic density (PSD). This is a specialized structure that contains all the critical proteins for synaptic transmission and plasticity and it is essential for receptors trafficking and postsynaptic signal transduction [8]. Alterations in the composition of the PSD are associated with several synaptic defects and cognitive impairments [9]. Importantly, tetraspanins also play a role in membrane compartmentalization, associating with each other and other partners in highly ordered protein complexes called tetraspanins-enriched microdomains (TEM). These microdomains constitute a dynamic scaffold platform important for cell signal transduction and cell to cell communication [10,11]. The highly organized microdomains and the molecular processes occurring there could be easily compared with the PSD. In fact, Tspan7, another member from the tetraspanin family, have been previously described to form part of the PSD scaffold complex and interestingly, mutations in *Tspan7* gene have been associated with X-linked intellectual disability [12]. In the latter paper, the authors showed that Tspan7 interacts with PICK1 in the PSD complex, regulating AMPA receptors trafficking and hippocampal spine development *in vitro*. Given that Tspan6 and Tspan7 are highly homologous members from the Tspan family, sharing 59% of aminoacid identity, we hypothetized that both proteins could be playing similar roles regulating synaptic function.

In order to determine the role of Tspan6 in the brain and to investigate the functional consequences of its deletion, we obtained a constitutive *Tspan6* knock out mouse model and we performed an electrophysiological, behavioral, biochemical and morphological characterization of the adult Tspan6 KO mice compared with wild type littermates.

## Materials and methods

### Tspan6 knock out mice and genotyping

**Fig 1:**
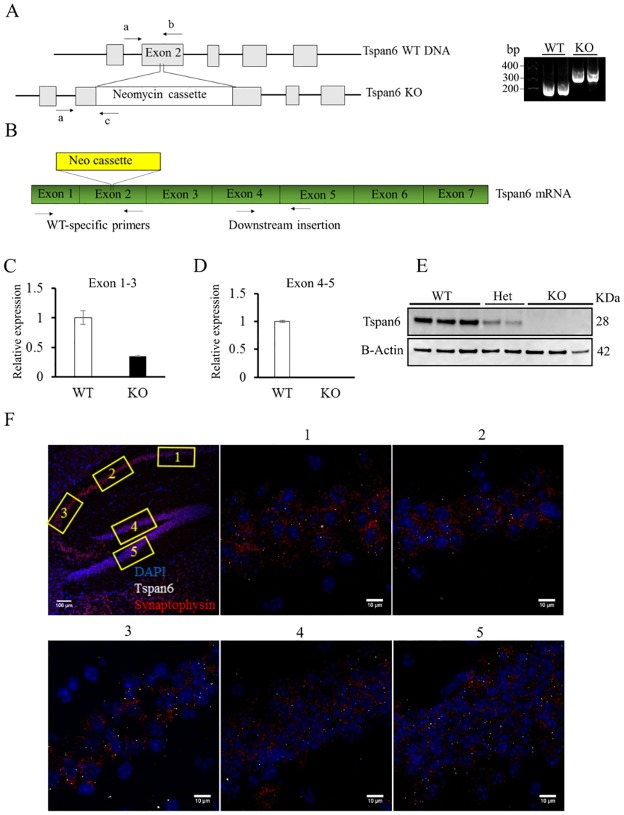
Generation of Tspan6 KO mice and Tspan6 expression in the brain. **(A)** The *Tspan6* KO mouse was generated by insertion of a neomycin cassette in the exon 2 of the *Tspan6* gene. Right panel show a representative agarose gel electrophoresis with the PCR products amplified with specific primers (a, b and c, shown by arrows in the left panel). **(B)** RNA was extracted from *Tspan6* KO and WT animals. Primers were designed between exon 1 and 3 (WT-specific primers), and exon 4 and exon 5 (primers downstream insertion). **(C)** Real time semi-quantitative PCR shows no RNA amplification between exon 1 and 3 in *Tspan6* KO mice due to the insertion of the neomycin cassette. **(D)** RNA amplification downstream the insertion is reduced in *Tspan6* KO mice (0.35± 0.01 mean fold change compared to WT) suggesting RNA degradation. Histogram shows mean (±S.E.M) fold changes normalized against WT expression, using either WT-specific primers (C) or primers downstream the insertion (D). Two housekeeping genes (Actin and GAPDH) were used for the normalization of the expression. **(E)** Neuronal lysates from cortical primary cultures from *Tspan6* WT, heterozygous and KO mice show the absence of Tspan6 protein in the KO condition. **(F)** RNA scope shows expression of Tspan6 RNA in the pyramidal layer of the hippocampus and granule cells from the dentate gyrus. First panel is a general view of the hippocampus (scale bar = 100μm). Panels 1 to 5 show box section in higher magnification (scale bar = 10μm).White dots are Tspan6 RNA molecules, synaptophysin RNA is stained in red and DAPI in blue.

This work have been approved by the ethical committee from the animal research center from the KU Leuven with a project number: P066/2014. Mice were euthanized by cervical dislocation for the collection of the tissue, and in case of signs of suffering. The *Tspan6* KO was generated by the Velocigene pharmaceutical company by the insertion of a Neomycin cassette in exon 2 of *Tspan6* gene. ES cells derived from the 129/OlaHsd mouse sub-strain were used to generate chimeric mice. F1 mice were generated by breeding with C57BL/6 females. F2 homozygous mutant mice were produced by intercrossing F1 heterozygous males and females. The KO line has subsequently been backcrossed several times to C57BL/6. For genotyping, tails were lysed with KAPA2G Fast HotStart Genotyping Mix (Sopachem) following the instructions from the company. For the PCR amplification 3 different primers were used (see Fig 1A). Primers sequence listed in Table 1.

**Table 1:** Primers sequence for PCR amplification.

Reverse common WT and KO (a)	5’- CTTACTCACCAGTTTCAGCATCCAG-3’
Forward WT-specific (b)	5’- TGTGATCAAGGACTCAAGCTTGTAC-3’
Forward KO-specific (c)	5’- GGGTGGGATTAGATAAATGCCTGCTCT -3’

### RNA extraction and real time semi-quantitative PCR

For RNA extraction, brain tissue was homogenized in 1ml of Trizol (Invitrogen, Life Technologies Corporation) using a syringe needle. After the addition of 200 μl of chloroform, samples were centrifuged and the aqueous phase was mixed with 1.25 volumes of ethanol 100%. Solution was transferred to miRVana spin columns (Ambion, Life Technologies Corporation) and washing and elution steps were done following the protocol described by manufactures. Reverse transcription of 200 ng RNA was performed using the Superscript II reverse transcriptase (Invitrogen, Life Technologies Corporation). Real time semi-quantitative PCR was performed using the LightCycler 480 Sybr Green (F. Hoffmann-La Roche Ltd). Cp (crossing points) were determined by using the second derivative method, and were normalized by two housekeeping genes (Actin and GAPDH). Fold changes were calculated with the ΔΔCt method [13]. The primer sequences used are listed in Table 2.

**Table 2:** Primers sequence for real time semi-quantitative PCR amplification.

Exon 1–3 forward	5’ TCGGAGGCTGCAGACTA 3’
Exon 1–3 reverse	5’ CATCGCGTACAGTTCAGCAT 3’
Exon 4–5 forward	5’ GTACAACTCCACAGGAGACT 3’
Exon 4–5 reverse	5’ CTCTCTGTGGATAACAGCC 3’
Actin forward	5’ TCCTCCCTGGAGAAGAGCTA 3’
Actin reverse	5’ GCAATGATCTTGATCTTC 3’
GAPDH forward	5’ TTGATGGCAACAATCTCCAC 3’
GAPDH reverse	5’ CGTCCCGTAGACAAAATGGT 3’

### RNA scope

For the RNA *in situ* hybridization we used the RNA scope multiplex fluorescent assay (Advanced Cell Diagnostics). Specific probes against Tspan6 and Synaptophysin were designed by the company and protocol described by manufactures was followed. Briefly, fresh frozen brains from *Tspan6* WT mice were cut in 10 μm sagittal slices with a cryostat. Sections were then postfixed with 4% PFA for 15 minutes, dehydrated with increasing ethanol concentration and incubated with Pretreat 4 solution for 30 minutes in a HybEZ^™^ Slide Rack. After that, sections were incubated with the appropriate probes for 2 hours at 40°C, and washed with washing buffer. Amplification was done by incubation at 40°C with AMP-FL (1–4) buffers for 30, 15, 30 and 15 minutes respectively, with two times 2 minutes wash in between steps. Finally, sections were incubated with DAPI for 30 minutes and slices were mounted and stored in the dark at 4°C. Pictures were acquired with a Leica SP8 confocal microscope, with the oil immersion 63x objective and maximal projections from 4 different z stacks are shown.

### Hippocampal primary cultures

Hippocampal neurons were obtained from E18 mouse embryos as previously described [14]. Briefly, embryonic hippocampi were isolated in Hank’s balanced salt solution (HBSS) with 10 mM HEPES, pH 7.4 and 0.25% trypsin and incubated for 15 minutes at 37°C. Trypsin was eliminated by washing three times with HBSS, and tissue was homogenized by pipetting. Homogenized neurons were plated onto 1mg/ml poly-lysine and 5 μg/ml laminin-coated 15 mm cover slips containing plating medium (MEM, 20% Glucose and 10% horse serum, Invitrogen) and incubated for 1.5 hours at 37°C and 5% CO2. After that, the medium was changed to Neurobasal/B27 medium (Invitrogen).

### Neuronal transfection and immunostainings

For the morphological studies, hippocampal primary neurons were transfected at DIV7 with 0.4 μg of pEGFP under de CMV promoter using Lipofectamine 2000 transfection reagent (Life technologies #11668–019). At DIV14, neurons were fixed with 4% paraformaldehyde for 10 minutes at room temperature, washed three times with PBS and permeabilized with 0.1% triton X-100 for 5 minutes. Blocking was done with 2% BSA, 2% fetal bovine serum and 0.2% gelatin for 30 minutes at room temperature. Then fixed neurons were incubated with the primary antibody (Chicken GFP, AVES, 1/1000) for 2 hours at room temperature, washed 3 times with PBS, and incubated for 1 hour with the secondary antibody (Alexa-488 anti-chicken, Invitrogen, 1/1000). After washing, cover slips were mounted with mowiol.

For the GluA1 surface expression, live 14 DIV hippocampal primary neurons were incubated for 10 minutes with anti GluA1 N-terminal antibody (gift from Dr. Andrew Irving, University of Dundee [15], 1/300) diluted in the conditioned Neurobasal medium from the neurons. Later, cells were fixed with 4% paraformaldehyde for 15 minutes at room temperature, washed three times with PBS and permeabilized with 0.25% triton X-100 for 5 minutes at 4°C. After 1 hour blocking with 10% BSA at 37°C, neurons were incubated over night with the secondary antibody (Alexa-555 anti-sheep, Invitrogen, diluted 1/1000 in 3% BSA). Next day, cover slips were washed with PBS and blocked again for 1 hour with 10% BSA at 37°C. Incubation with primary antibodies (MAP2, V-Glut1, Millipore, diluted in 3% BSA) was done for 2 hours at 37°C. Then neurons were washed with PBS and incubated for 1 hour with the secondary antibodies (Alexa-488 anti-rabbit, Invitrogen, Alexa-647 anti-guinea pig, Jackson ImmunoResearch, diluted 1/1000 in 3% BSA), washed again with PBS and mounted with mowiol.

Images were taken with confocal microscopy (Leica TCS SP5) and analyzed manually with ImageJ.

### Synaptosomes purification

11-month old *Tspan6* KO mice and wild type littermates were sacrificed by decapitation and hippocampi were dissected in cold PBS. Synaptosomes were obtained using Percoll gradients as previously described [16]. Briefly, one hippocampus was homogenized in 2 ml of 0.32 M sucrose, 1 mM EDTA, 1mg/ml BSA and 5 mM HEPES pH 7.4 with a Glass-teflon douncer at 245 rmp and 4°C. 400 μl from the homogenate were kept for total input. The rest was spun down for 10 minutes at 3000 G at 4°C. The supernatant was spun down again for 12 minutes at 14000 G. After that, the pellet was resuspended in 220 μl of Krebs-Ringer (40 mM NaCl, 5 mM glucose, 1 mM EDTA and 10 mM HEPES pH 7.4) and 180 μl of Percoll^®^ (45%v/v). After mixing properly, samples were spun down at 14000 rpm for 2 minutes. The enriched synaptosomal fraction (on the surface of the solution) was recovered and resuspended in 1 ml of Krebs-Ringer buffer. Finally, synaptosomes were recovered by centrifugation during 30 seconds at 14000 rpm and the pellet resuspended in 50 μl of 10 mM HEPES, 1 mM EDTA. Protein concentrations were quantified with the Pierce^®^BCA Protein Assay kit (Prod #23227).

### PSD isolation

Protocol slightly modified from [17]. Briefly, synaptic pellet was resuspended in 200 μl of cold 0.1 mM CaCl2 and then 200 μl of 2x solubilization solution pH 6 (40 mM Tris, 2% Triton X-100) was added. After 20 minutes incubation at 4°C with mild agitation, samples were centrifuged for 30 minutes at 40000 x g and the pellet was resuspended in 50 μl of 1x solubilization solution pH 8 (20 mM Tris, 1% Triton X-100). Then, samples were incubated again for 20 minutes at 4°C with mild agitation and centrifuged for 30 minutes at 40000 x g. Finally, the pellet containing the post-synaptic densities was resuspended in 20 μl of 0.2% SDS in PBS with protease inhibitors, and the supernatant containing the pre-synaptic proteins was kept and stored at -20°C. Protein concentrations were quantified with the Pierce^®^BCA Protein Assay kit (Prod #23227).

### Western blot and antibodies

Proteins separated by SDS-PAGE (NuPAGE^®^ Novex 4–12% Bis-Tris gel; Invitrogen) and transferred to a 0.2 μm nitrocellulose membrane, were probed with specific antibodies [Mouse CamKIIα (Santa Cruz, 1/10000), Chicken mGluR5 (AVES, 1/1000), Mouse PSD-95 (BD Biosciences, 1/500), Mouse GLT-1 (Abcam, 1/250), Mouse β-actin (Sigma, 1/5000), Rabbit GluA1 (Oncogen, 1/1000), Rabbit GluA1 pSer831 (Sigma, 1/1000), Rabbit GluA1 pSer845 (Millipore 1/5000), Mouse GluA2 (Calbiochem/Milipore, 1/500), Rabbit NR2A (Milipore, 1/500), Mouse NR2B (Acris, 1/1000), Rabbit Tspan7 (Santa Cruz, 1/500), Rabbit Tspan6 (Abgent, 1/500)]. Immunodetection was done with horseradish peroxidase-coupled secondary antibodies (Bio-Rad, 1/5000) and the chemiluminescent detection reagent Renaissance (PerkinElmer Life Sciences).

### Golgi staining and spine morphology

10 months-old *Tspan6* KO and wild type littermates were transcardially perfused with 4% PFA in PBS, and brains were processed with FD Rapid GolgiStain kit (PK401; FD NeuroTechnologies) according to manufacturer instructions. Stained brains were embedded in 4% low melting agarose and cut with vibratome at 100 μm. Imaging was performed with a Zeiss Axioplan2 upright microscope with 100x Plan-Apochromat oil immersion (NA = 1.4) objective. Each image was a z-series of images and the obtained stacks were directly analyzed using IMARIS (Bit-plane AG, Zurich, Switzerland). Morphology of Golgi stained neurons was quantified based on the reconstructions computed by IMARIS.

### Electrophysiology

Slices of 400 μm-thickness were obtained from 11 month-old *Tspan6* KO mice and control littermates and maintained in artificial cerebrospinal fluid (ACSF), saturated with 95% O_2_/5% CO_2_, at 32°C for 60 minutes. ACSF composition: 119 mM NaCl, 2.5 mM KCl, 1 mM NaH_2_PO_4_, 11 mM glucose, 2.5 mM CaCl_2_, 1.2 mM MgCl_2_, 26 mM NaHCO_3_. After 1 hour recovery, slices were placed in the recording chamber under ACSF perfusion in the presence of 0.1 mM picrotoxin to block GABA_A_ receptor-mediated inhibitory transmission. Field excitatory postsynaptic potentials (fEPSPs) were recorded with glass electrodes (filled with ACSF, 0.2–0.8 MΩ) placed in the apical dendritic layer of CA1 area. Paired pulse facilitation experiments were done with inter-stimulus interval of 50, 100, 200 and 400 milliseconds. LTP was induced with a theta-burst stimulation protocol (TBS): 5 trains of 10 bursts at 5 Hz, each burst consisting of 4 pulses at 100 Hz. LTD was induced with 1 Hz, 900 pulses Schaffer collateral stimulation. Electrophysiological recordings and data acquisition were performed with Multiclamp 700A/B amplifiers (Molecular Devices). Data acquisition and analysis was done with pClamp software.

### Behavioral tests

11 months-old male mice were housed in standard mouse cages (3–5 mice per cage) with wood-shaving bedding. Food and water were available *ad libitum* in temperature and humidity controlled rooms with a 12-hour light-dark cycle. All behavioral experiments were performed during the light phase of their cycle and approved by the institutional ethical committee of the KU Leuven for use on experimental animals.

#### 24h spontaneous activity

Mice were housed individually in small mouse cages for 24 hours with free access to food and water. During the test period (4 pm-4 pm), the activity of the mouse was registered by three infrared beams situated outside the cage. The established day-night light cycle was maintained (lights off at 8pm- lights on at 8am).

#### Motor coordination and balance on the Rotarod

Animals had to balance on a rotating beam at increasing rotation speed. After 2 days of training at constant (day 1: 4 trials 4rpm/2minutes) and increasing speed (day 2: 4 trials of 4rpm/50s and accelerating to 8rpm/70s), mice were tested for balancing on a rotating beam (4-40rpm in 5 minutes, with a cut-off of 5 minutes), with inter-trial intervals of 30-60-30 minutes. Latency to fall was recorded.

#### Open field exploration

Locomotor activity was measured using a 50 cm × 50 cm arena (transparent Plexiglass), illuminated by 2 bright spots. Mice were habituated for 30 minutes in the dark and placed in the illuminated arena to explore for 10 minutes. Movement of the mice was recorded and analyzed using EthoVision® video tracking software (Wageningen, the Netherlands). To assess exploration, total path length, speed, and corner and center crossings were recorded.

#### Anxiety-related exploration in elevated plus maze

The elevated plus maze consisted of two open arms (21 cm × 5 cm) and two enclosed arms (21 cm × 5 cm) with high walls. The same type of arms were located opposite to each other. The maze was elevated to a height of 30 cm. Animals were placed in the center of the maze for 5 minutes of free exploration. During the test period, the activity of each mouse was recorded and the time spent in the open arms was quantified manually.

#### Spatial learning in the Morris water maze

Protocol previously described in [18]. Briefly, a circular pool (150 cm diameter), filled with water (26 ± 0.5°C, opacified with nontoxic white paint) was virtually divided into 4 equivalent quadrants. An escape platform (15 cm diameter) was placed at a fixed position in one of the quadrants 0.5 cm below the water surface. For acquisition of spatial memory, mice were trained for 10 days (4 trials/day; 15–30 minutes inter-trial interval) to find a hidden platform starting randomly from 4 different positions. Probe trials were done on day 6 and day 11by removing the platform and the search pattern of mice was recorded for 100s. Swim paths were recorded using EthoVision^®^ video tracking (Noldus, Wageningen, the Netherlands), and parameters such as escape latency, distance traveled and swim velocity, and number of platform crossings were extracted.

#### Contextual and auditory fear conditioning

Context- and cue-dependent fear conditioning was studied using a protocol adapted from [19] and described earlier [20]. Behavioral freezing was quantified as reliable readout for innate and acquired fear in rodents. Briefly, on the first day, mice were placed for 5 minutes in the StartFear cage (Panlab, Spain) in a specific context (dark environment, grid floor, ethanol odor) for acclimation. On the second day, mice were replaced in the same context and after 2 minutes of exploration (baseline), twice a 30s tone (4 kHZ, 80 dB) was delivered that co-terminated with a 2 s shock delivered through the grid floor (0.3 mA). Inter-stimulus interval was 60 s. After the second tone-shock presentation, mice were returned to their home cage. Later, animals were returned to the testing chamber (same context) for 5 minutes (context trial). After 90 minutes spent in their home cage, mice were tested again for cue-dependent memory in a novel context (illuminated environment, different odor, white plastic covering the grid floor) for 6 minutes. After the 3 minutes of exploration (new context), the tone was delivered for 3 minutes (Tone test). During each trial, freezing behavior was recorded by a pressure sensitive weight transducer system (Panlab, Spain). The percentage of freezing was calculated per trial.

## Results

### *Tspan6* KO generation and expression in the brain

*Tspan6* constitutive KO mouse was generated by insertion of a Neomycin cassette in the exon 2 of the *Tspan6* gene. To validate the KO model, we first purified DNA from *Tspan6* KO mice and wild-type controls and we amplified it by PCR using a common primer upstream the insertion, a WT-specific primer against exon 2, and a KO-specific primer against the Neomycin cassette (Fig 1A), demonstrating the specificity of the insertion in exon 2. To further validate the KO model we extracted RNA from *Tspan6* KO brains, we retrotranscribed it into cDNA and we run a real time semi-quantitative PCR. For the cDNA amplification we used primers against exon 1 and 3, flanking the insertion site in exon 2. The Neomycin cassette is more than 6000 base pairs long and the amplification cycle is only 40 seconds, so if the cassette is inserted at the RNA level, we should not see any amplification in the tissue from KO animals. Indeed, this is the case as shown in Fig 1C. When we amplified cDNA using primers downstream the insertion (in exon 4 and 5), we could detect some expression of Tspan6 RNA in KO animals, but this expression was reduced more than 60% compared with wild-type controls (0.35±0.01 relative expression normalized to WT) showing that in the KO tissue, Tspan6 RNA is being degraded due to the insertion of the cassette (Fig 1D). We also analyzed the levels of Tspan6 protein by western blot in neuronal lysates from cortical primary cultures confirming a decreased amount of Tspan6 protein in the heterozygous mice and a total absence of protein in the KO condition.

We then studied the expression pattern of Tspan6 in the brain. Mouse brain atlas showed expression of Tspan6 mRNA predominantly in the hippocampus, hypothalamus, midbrain, pons, medulla and cerebellum. To confirm the expression of Tspan6 in the hippocampus, we performed a RNA scope experiment using specific probes against Tspan6 (white dots, Fig 1E) and synaptophysin (red dots, Fig 1E) RNA. As shown in Fig 1E, we could confirm expression of Tspan6 in the hippocampal pyramidal layer (CA1-CA3) and in the granule cells from the dentate gyrus.

### *Tspan6* deletion does not affect hippocampal spine number and maturation *in vitro* and *in situ*

Spine formation and maturation are essential processes for a correct brain function, and deficits in spine development have been linked to several cognitive diseases [21,22]. In fact, Tspan7, homologous to Tspan6 and associated with X-linked intellectual disability, has been described to play a role in synapse development *in vitro* [12].

**Fig 2:**
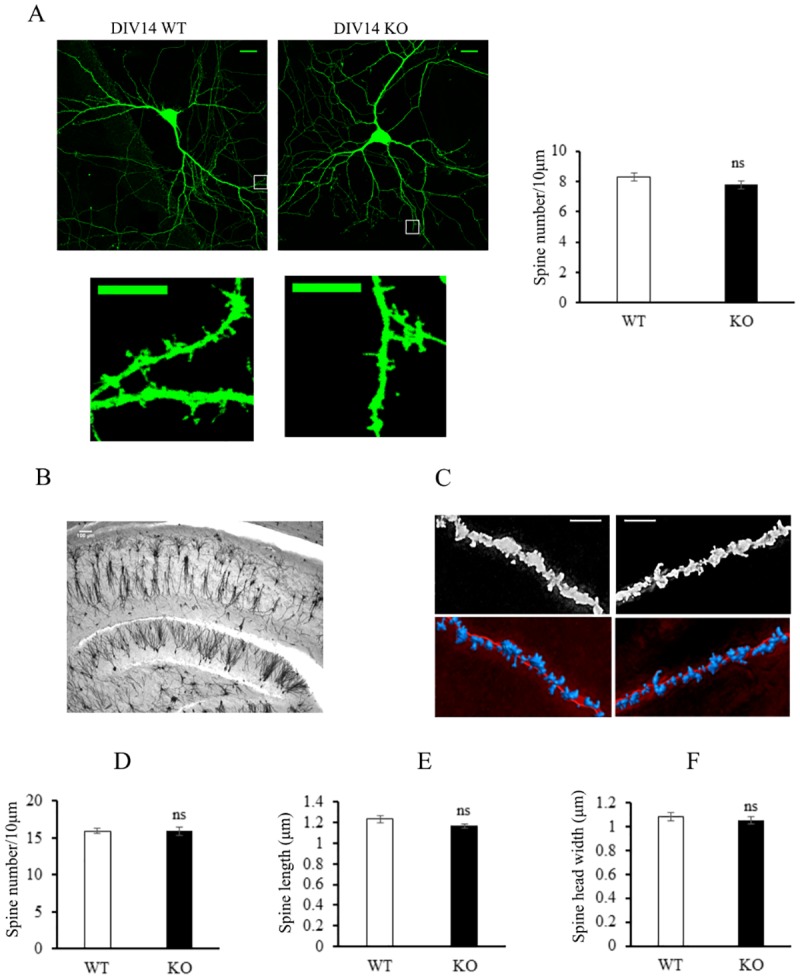
Tspan6 does not affect synapse formation and maturation *in vitro* and *in vivo*. **(A)**Representative images from WT and *Tspan6* KO hippocampal primary neurons transfected with EGFP and fixed after 14 days *in vitro* (scale bar = 20 μm). Right panels show box sections in higher magnification (scale bar = 10μm). 15 neurons and more than 90 dendritic sections were examined from 3 independent cultures. Only spines more than 80μm from the soma were analyzed. Histograms compare mean (±S.E.M) filopodia and spine density (number/10μm of dendrite). **(B)** Golgi staining of 100 μm coronal sections from 10 month-old *Tspan6* KO and WT mice. Scale bar = 100 μm. **(C)** Representative images of dendritic spines from basal secondary dendrites from CA1 hippocampal neurons and the IMARIS reconstruction to analyze spine morphology. Scale bar = 5μm. Histograms compare mean (±S.E.M) spine density (number/10μm of dendrite) **(D)**, length **(E)** and head width **(F)** between Tspan6 KO and WT mice. *n* = 19 to 22 neurons from 3 different mice.

To address whether Tspan6 plays a role in synaptic maturation *in vitro* we cultured hippocampal primary neurons derived from E18 *Tspan6* KO and control littermate embryos. Cells were transfected with EGFP at *day in vitro* (DIV) 7, and fixed at DIV 14. At this time *in vitro* neurons present mature morphological and functional synapses, reflected by the presence of high spine density and spontaneous electrical activity [14]. Quantification of density of synaptic spines (number/10μm) in dendrites more than 80μm away from the soma revealed no differences between the two groups (WT: 8.3±0.3 versus KO: 7.8±0.2; *p*>0.05, T-test) (Fig 2A).

We next evaluated the consequence of *Tspan6* knockout *in situ* in the brain. To this end, we performed Golgi staining in adult brains from 10 months-old *Tspan6* KO and control littermates. We focused the study on hippocampal CA1 pyramidal neurons as Tspan6 is highly expressed here, and changes in spine density in this brain region have been associated with cognitive diseases such as Alzheimer disease [23] and Rett syndrome [24]. Basal secondary dendrites from CA1 pyramidal neurons were analyzed with IMARIS software, allowing 2D reconstruction (Fig 2B and 2C). Similarly to what was observed in the *in vitro* studies, both *Tspan6* KO mice and WT littermates show comparable number of spines per 10μm dendrite (Fig 2D).

Next, we investigated whether the lack of *Tspan6* could have an effect on the maturations of dendritic spines. Longer and thinner spines (filopodia-like) are considered less mature, and an increased proportion of these type of spines and average length has been associated with neurological disorders [25]. Analysis of spine length and head width in *Tspan6* KO and WT pyramidal neurons revealed similar values in both groups (Fig 2E and 2F).

Altogether these first series of results suggest that the lack of Tspan6 does not result in major adverse changes in morphological synapse formation and maturation.

### Tspan6 regulates basal synaptic transmission and LTP

**Fig 3:**
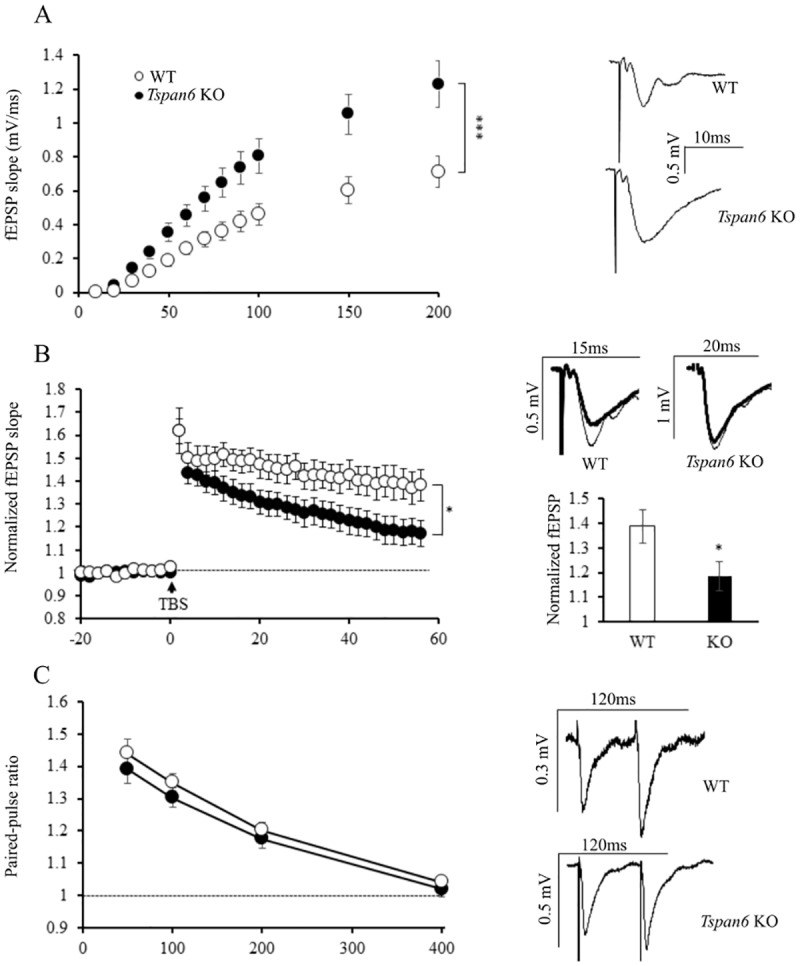
*Tspan6* KO mice show an increased basal synaptic transmission and impaired LTP in the CA3-CA1 synapses of the hippocampus. **(A)** Input-output relations between stimulus intensity applied to the Schaffer collateral fibers and slope of field excitatory postsynaptic potentials (fEPSP) recorded in the *stratum radiatum* of CA1. Right panel show representative traces of fEPSP at 90μA stimulation from Tspan6 KO and control acute slices. *n* = 30 WT and 25 *Tspan6* KO slices from 9 to 12 different mice per group. (F _(11,583)_ = 9.447, p<0.0001, Repeated measurements ANOVA). **(B)** LTP was induced in CA1 neurons by theta-burst stimulation (5 trains, each with 10 bursts at 5 Hz, each burst containing 4 pulses at 100 Hz) and fEPSP slope is normalized to 20 minutes baseline. Insets: representative traces averaged from the baseline (thick lines) or from the last 10 minutes of the recordings (thin lines). Histogram compares mean (±S.E.M) normalized fEPSP slope from the last 10 minutes of the recordings from *Tspan6* KO and control slices (*p* = 0.026, T-test). Between 10 and 14 slices were analyzed from 9 different mice per group. **(C)** Paired-pulse facilitation ratios evoked by stimulation of the Schaffer collateral fibers with different interstimulus intervals (50, 100, 200 and 400ms). Representative traces of the paired-pulse facilitation at 50ms interstimulus interval from WT and KO slices are shown in the right. 27 slices from 8 to 10 different mice were analyzed per group.

To study the potential role of Tspan6 in synaptic transmission we performed electrophysiological recordings in acute slices from adult *Tspan6* KO and control littermate mice. First, input-output experiments were carried out to evaluate a possible effect on basal synaptic transmission. We measured the slope of field excitatory postsynaptic potentials (fEPSP) recorded from the *stratum radiatum* of CA1 as a function of stimulation intensity applied to the presynaptic Schaffer collaterals fibers from CA3. The input-output curve from *Tspan6* KO acute slices (*n* = 25) was significantly larger compared to the WT littermates (*n =* 30) at any given stimulus intensity (F _(11,583)_ = 9.447; p<0.0001, repeated measures ANOVA) (Fig 3A), implying that Tspan6 plays an important role in synaptic functionality that is not associated to overt structural alterations of the synapse (see above). To restrict the locus of the observed changes, all the recordings were done in the presence of picrotoxin, an inhibitor of the GABA_A_ receptor-mediated currents. In this manner we can conclude that the enhanced basal synaptic transmission shown in *Tspan6* KO mice is not due to a reduced inhibition, but a true increased in excitatory transmission.

In order to substantiate the functional relevance of Tspan6, we next investigated the effect of its deletion in a long-term synaptic plasticity paradigm: long term potentiation (LTP). We induced LTP by presynaptic theta-burst stimulation of the Schaffer collateral fibers. WT slices (*n =* 9) yielded significantly more potentiation than *Tspan6* KO slices (*n =* 14) 60 minutes after stimulation (WT: 1.4±0.07 versus KO: 1.18±0.06 times over the baseline, *p*<0.05, T test) (Fig 3B). These data confirm that Tspan6 is an important regulator of synaptic function, both at basal conditions and during synaptic plasticity.

On the other hand, long-term depression (LTD) was not affected in *Tspan6* KO slices (S1 Fig), suggesting that the alterations in plasticity are specific for LTP. To determine whether the observed effects in basal synaptic transmission and LTP were due to changes in the presynaptic terminals of the *Tspan6* KO neurons, we performed paired-pulse facilitation (PPF) experiments. Fig 3C shows that PPF ratios are similar between WT and KO slices, suggesting that the increase of basal synaptic transmission and impaired LTP in Tspan6-deficient neurons are not due to alterations in presynaptic function.

### Levels of hippocampal synaptic proteins are not changed in *Tspan6* KO brains

**Fig 4:**
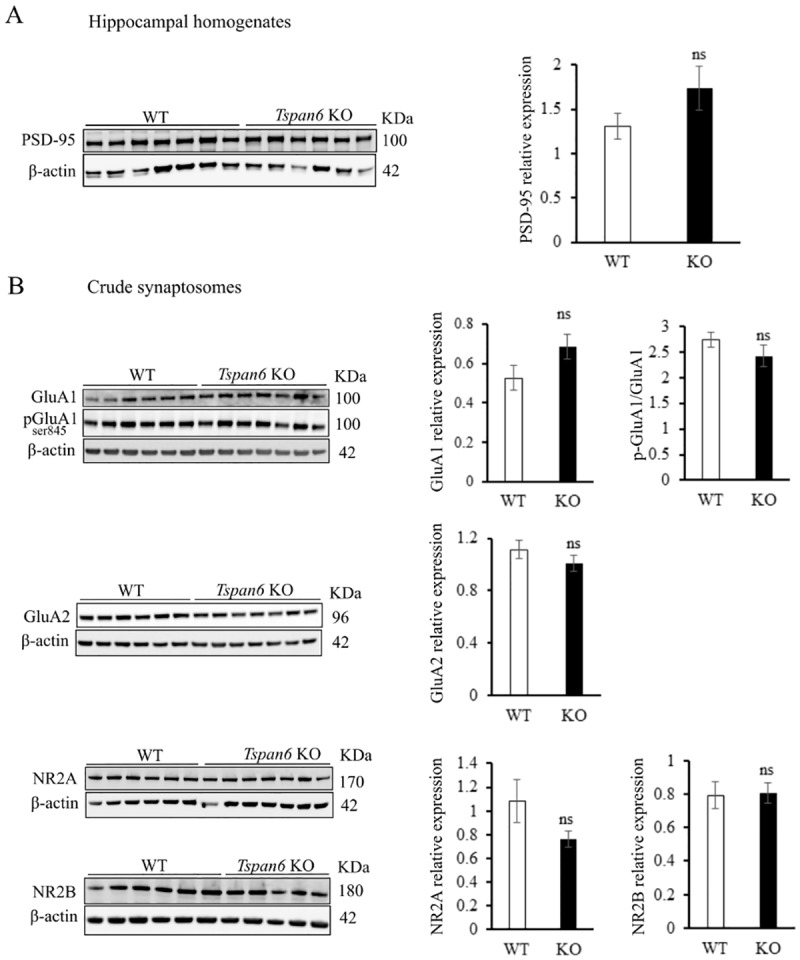
No changes in synaptic markers in the hippocampus from *Tspan6* KO mice. **(A)** Levels of synaptic markers are not altered in hippocampal homogenates from *Tspan6* KO mice compared with controls (n = 7 mice per group). **(B)** Crude synaptosomes were isolated from 10 month old *Tspan6* KO (*n* = 7) and control (*n* = 6) hippocampus and levels of AMPA and NMDA receptor subunits were analyzed by western blotting. Histograms compare mean (±S.E.M) relative expression normalized to loading control (β-actin).

The previous data show that *Tspan6* deletion affects synaptic transmission possibly at the postsynaptic compartment. We speculate that the increased transmission could be a consequence of an increased number of synaptic contacts in *Tspan6* KO hippocampi. To explore this question, we homogenized hippocampi from 11 months-old *Tspan6* KO and control littermates and we quantified the levels of postsynaptic markers by western blotting. As shown in Fig 4A no significant differences in PSD95 levels were detected between *Tspan6* KO and WT hippocampi. This result is also consistent with the similar spine density in WT and *Tspan6* KO neurons (Fig 2).

Changes in synaptic transmission can also be explained by an increased response to glutamate, the main excitatory neurotransmitter in the brain. The normal PPF showed in Fig 2C suggests no alterations in the presynaptic release probability in *Tspan6* KO hippocampus, therefore we hypothesized that the increased glutamate response could be a consequence of an increase number of glutamate receptors in the postsynaptic terminal. To test this hypothesis, we isolated hippocampal synaptosomes from 11 months-old *Tspan6* KO and WT littermates and compared the synaptic levels of different subunits of AMPA, NMDA and mGluR receptors. No significant differences were observed between *Tspan6* KO synaptosomes compared WT (Fig 4B, S3D Fig). To test whether differences in glutamate receptors could be restricted to the postsynaptic region, we analyzed the levels of the same subunits on purified postsynaptic densities (PSD) from hippocampal synaptosomes. This analysis did not reveal significant differences in the postsynaptic levels of AMPA or NMDA receptors in *Tspan6* KO mice compared to wild types (S3A Fig).

**Fig 5:**
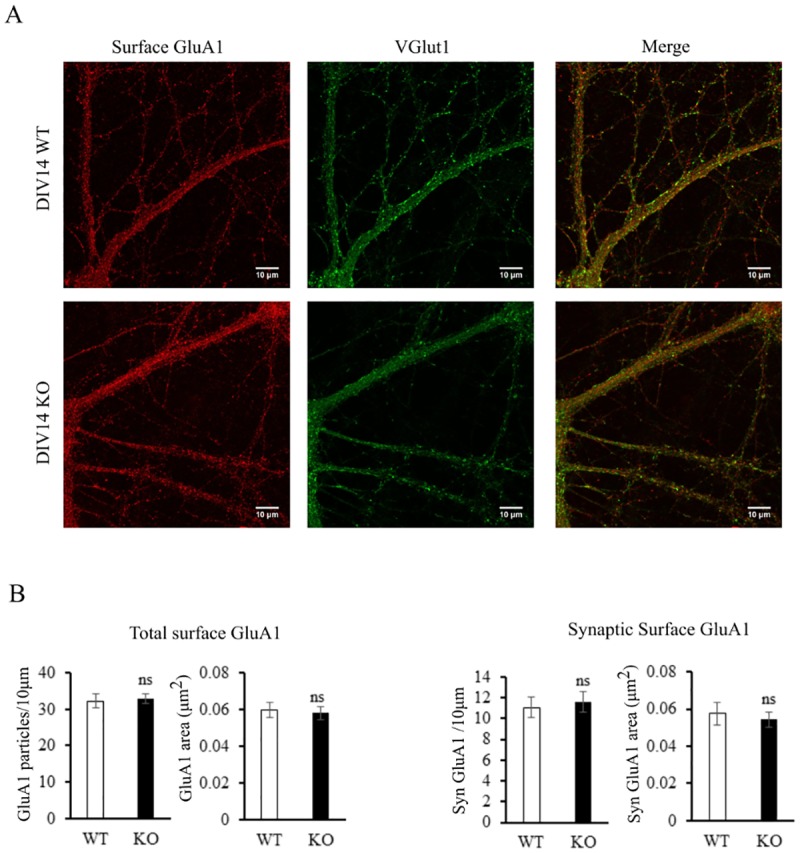
Surface expression of GluA1 is not increased in *Tspan6* KO hippocampal primary neurons. **(A)** Representative images from 14 days *in vitro* WT and *Tspan6* KO hippocampal primary neurons stained with an extracellular N-terminal domain GluA1 antibody before fixation (surface expression), and with VGlut1 antibody after fixation. **(B)** Histograms comparing mean (±S.E.M) density **(**particles/10μm) and area (μm^2^**)** from total cell surface GluA1 particles. **(C)** Histograms comparing mean (±S.E.M) density **(**particles/10μm) and area (μm^2^**)** from synaptic surface GluA1 particles. Scale bar = 10μm. *n* = 28 (WT) and 31 (*Tspan6* KO) neurons from 4 different embryos, 2 independent cultures.

It is important to mention that none of the previous assays discriminate between intracellular and cell-surface localization of the receptors. To address this question, hippocampal neurons in culture from *Tspan6* KO and WT animals were incubated with a GluA1-specific antibody that recognizes the extracellular N-terminal domain of the subunit before fixation [15]. We focused specifically on the GluA1 subunit of AMPA receptors because induction of LTP is thought to involve the transient incorporation of GluA2-lacking AMPA receptors [26]. For this reason, an increase in the cell surface expression of GluA1 in the KO condition would explain the observed impaired LTP and the enhanced basal transmission. However, neither the density of surface GluA1 particles (WT: 32.23± 1.93; KO: 32.81± 1.3 particles/10μm; *p*>0.05, T-test) nor the area (WT: 0.059±0.004; KO: 0.058±0.003 μm^2^, *p*>0.05, T-test) were significantly changed in the *Tspan6* KO neurons (Fig 5B). We also analyzed the co-localization of surface GluA1 with the synaptic marker VGlut1, to discriminate between synaptic versus non-synaptic GluA1 population. Again, the density (WT: 11.07 ± 0.95; KO: 11.6 ± 0.96 particles/10μm, *p*>0.05, T-test) and the area (WT: 0.058 ± 0.006; KO: 0.045 ± 0.004 μm^2^ *p*>0.05, T-test) of synaptic GluA1 were not significantly altered in *Tspan6* KO primary neurons (Fig 5C).

To explore the possibility of a defect in glutamate uptake by astrocytes, we evaluated the levels of the glutamate transporter GLT-1 in hippocampus homogenates, however, no significant differences were found between *Tspan6* KO and wild-type mice (S3E Fig).

As synaptic plasticity was affected upon *Tspan6* deletion, we also investigated whether basic molecular mechanisms associated to LTP were affected in *Tspan6* KO mice. Phosphorylation of AMPA receptors in the brain plays a key role in the regulation of their function and trafficking and it is important for the expression of synaptic plasticity. Particularly, phosphorylation of Serines 831 and 845 of the GluA1 subunit, are believed to regulate single-channel conductance [27] and open channel probability [28], respectively, and both are important for LTP induction. We therefore analyzed the levels of phosphorylation of GluA1 in these residues under basal conditions. These experiments did not reveal significant differences in the phosphorylation state of GluA1 Ser831 (Fig 4B) or GluA1 Ser845 (S3B Fig) in *Tspan6* KO synaptosomes compared to WT controls. Ca^2+^/Calmodulin-dependent protein kinase II (αCamKII) activation by phosphorylation is also essential for LTP induction and it is one of the kinases responsible for GluA1 phosphorylation on Ser831 [29]. We also examined the synaptic levels of phosphorylated CamKII (pCamKII) and we found similar values in *Tspan6* KO and control synaptosomes (S3C Fig).

### Adult *Tspan6* KO mice show normal locomotor behavior and normal hippocampus-dependent memory performance

To evaluate whether the changes in synaptic transmission have a functional effect in adult mice, we performed a number of behavioral studies in 11 months-old *Tspan6* KO mice (n = 8) and control (n = 6) littermates. We first analyzed the spontaneous cage activity by the number of beam-crossings during 24 hours. This test allows to assess general changes in activity, and circadian rhythms. Both genotypes showed similar activity profile during the dark and the light phase (Before night: F_(6,72)_ = 0.53; During the night: F_(24,288)_ = 1.09; Repeated measurements ANOVA) (S4A Fig).

We also investigated the exploratory behavior in the open field and tracked the trajectories over 10 minutes. We observed no difference in path length but a trend in increased walking speed in *Tspan6* KO mice compared to controls (WT: 0.044±0.004 versus KO: 0.053±0.003m/s; *p* = 0.07, T test). The number of entries to center and periphery were also comparable in both groups (S4B Fig). Similarly, the elevated plus maze, another test evaluating anxiety-like behavior, did not show differences in open arm time between the genotypes (S4C Fig).

**Fig 6:**
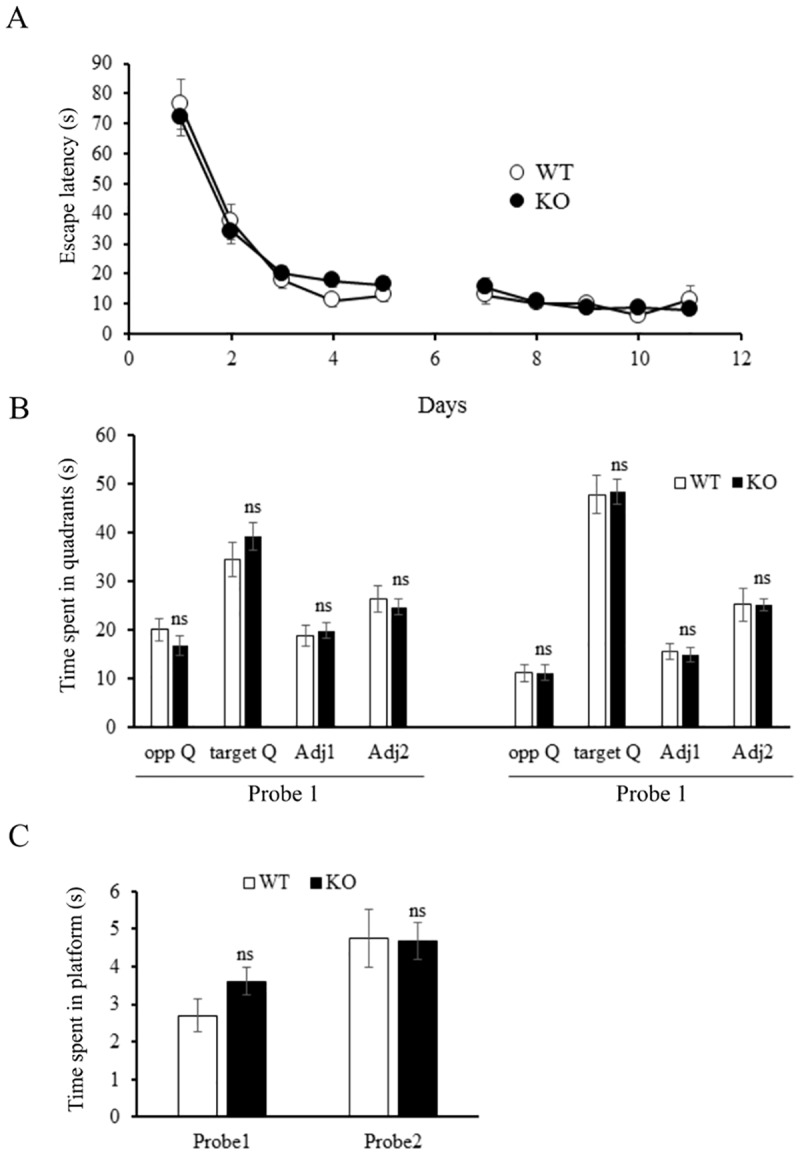
*Tspan6* mice show normal behavior in the Morris water maze test. Mice were trained in the hidden platform Morris water maze for 10 days (4 trials/day) and tested with a probe trial (100s) on day 6 and 11. **(A)** Curve shows no differences between *Tspan6* KO and WT mice in the learning-related decrease in escape latency (time required to find the platform) during the acquisition phase. **(B)** Probe trial performance illustrate spatial memory for the platform position as both groups show a preference for the quadrant where the platform was located during the training (target quadrant) with no significant differences between the groups. **(C)** Spatial learning was analyzed in more detail quantifying the time that the mice spend swimming in the specific area of the quadrant were the platform was during the training showing no changes in the KO animals compared to controls. *n* = 12 WT, 21 KO animals.

Long term synaptic plasticity is considered an essential molecular mechanism for learning and memory [30]. As *Tspan6* KO mice showed impairments in LTP, we investigated whether the electrophysiological impairment had a true functional counterpart, reflected in hippocampal-dependent memory processes. To address this possibility we first performed a contextual fear conditioning test. Both groups learned to associate a cue (tone) and a context with an aversive event (electric shocks), without significant differences (S5A Fig). Next, we analyzed spatial cognition in the Morris water maze. All mice learned to locate the hidden platform after 10 days of acquisition training, reflected by a decrease in escape latency (F_(9, 279)_ = 74.81; *p*<0.0001 RM-ANOVA, factor time) (Fig 6A). Similar to the open field, *Tspan6* KO mice showed a slightly increased swimming speed (WT: 18.4±3.5 versus KO: 19.9±4.5 cm/s; *p* = 0.04, T test) (data not shown). Two probe trials were performed after 5 and 10 days of training (probe trial 1 and 2, respectively), when the platform was removed and swim trajectories were tracked for 100s. As shown in Fig 6B, after 5 and 10 days of training, both groups showed a similar swimming preference for the quadrant where the platform was located. We also evaluated the accuracy of spatial reference memory by quantifying the time spent swimming at the defined platform position, finding no significant differences between the two genotypes (Fig 6C).

Finally, we evaluated motor learning and behavior with the rotarod test. *Tspan6* KO mice performed worse compared to controls during the first trials (F_(3,114)_ = 4.4, p<0.05, Repeated measurements ANOVA, time x genotype interaction) but not during the last ones, suggesting that *Tspan6* KO mice have a delayed motor learning without defects in the motor capacities (S5B Fig).

## Discussion

In this paper we have identified Tspan6 as a modulator of the synaptic transmission and plasticity machinery. *Tspan6* KO hippocampal slices showed an enhanced basal synaptic transmission in CA3-CA1 synapses and an impaired LTP. Although at first sight increased basal synaptic transmission and reduced LTP appear paradoxical, it is possible that in fact the impaired LTP is the consequence of potentiated post-synaptic membrane due to long-lasting (lifelong) enhanced basal transmission. In this scenario of constitutively potentiated post-synaptic membrane it is then natural that theta burst stimulation cannot induce further potentiation. This type of “occluded” LTP has been previously reported in conditions of overexpression of proteins that enhance synaptic transmission [31,32]. In addition, our results suggest that Tspan6-dependent potentiation of transmission share the same molecular mechanisms required for LTP. However, it still remains to be stablished how the absence of Tspan6 leads to an enhaced synaptic transmission without changes in the AMPA receptor surface expression or accumilations at PSD franction. One possibility may be linked to changes in biophysical properties of the AMPA receptor independent from GluA1 phosphorylation. For example, AMPA receptor association with transmembrane AMPA receptor regulatory proteins (TARPs) has been shown to modulate gating properties of the AMPA receptor ion channel [33]. However, most TARPs also modulate AMPA receptor surface expression [34] which we have not observed here.

As mentioned before, genomic deletions including the *Tspan6* gene have been described in two female patients with epilepsy and intellectual disability [3,5]. Both publications concluded that the pathology was due to deletions in the *PCDH19* gene, located in the same genomic region than *Tspan6*. However, deletions spanned more than 4 other genes including *Tspan6* and the possibility that these genes also contribute to the pathology has not been ruled out. Interestingly, increased CA1 excitability has been associated with epileptic activity in a mouse model of temporal lobe epilepsy [35]. Our observation that the lack of Tspan6 enhances CA1 excitability raises the possibility that the epileptic phenotype of the patients is partially due to *Tspan6* gene deletion. In addition, the impaired LTP observed in *Tspan6* KO mice could also support the cognitive defects shown in the patients.

However, in this study, despite the impaired LTP observed in the hippocampus from *Tspan6* KO mice, we found no significant deficits in hippocampal-dependent memory tasks (Morris water maze and contextual fear conditioning). While a functional link between *ex vivo* hippocampal LTP and hippocampus dependent behavior is often stipulated, there are several examples in the literature that do not support such an absolute correlation [36–39]. This suggests that learning and memory defects might be a consequence of a whole neuronal network dysfunction not limited to the hippocampus alone.

Some members of the tetraspanin family, including Tspan6, contain in the C-terminal domain, tyrosine-based sorting sequences that are putative PDZ bindings motifs [40]. Interestingly, the first PDZ-containing protein identified was the postsynaptic protein 95 (PSD95) [41] and PDZ-dependent interactions are essential for NMDA and AMPA receptors trafficking in the synapses and are the bases for synaptic transmission and plasticity [8]. In fact, Tspan7 forms part of the PSD scaffold complex interacting with the PDZ domain of PICK1 and regulating the trafficking of AMPAR through this interaction [12]. In the latter paper, authors suggested a positive role of Tspan7 in synaptic transmission, showing that Tspan7 downregulation in hippocampal primary cultures reduced excitatory synaptic function. Intriguingly, *Tspan6* deletion produce the opposite effect in hippocampal acute slices, enhancing basal transmission. These indications suggest that despite the high amino acid residue identity shared between Tspan6 and Tspan7 [42], both proteins may play opposite roles. We also considered that the enhanced synaptic transmission in the Tspan6 KO condition could be due to an increased expression of Tspan7 as a compensatory mechanism. Although we did not observe a significant upregulation of Tspan7 in hippocampal homogenates from *Tspan6* KO mice to explain the reported changes in synaptic transmission (see S2 Fig), we cannot rule out the possibility that the lack of molecular or behavioral phenotype showed in *Tspan6* KO mice might be due to a Tspan7 increase in discrete, functionally relevant, areas of the neuron.

Interestingly, in the recent years, several new proteins have been described to form part of the PSD complex [43,44] that were not previously identified in AMPA receptor interactome studies. [45] In addition, it has been recently shown that AMPA receptors are localized at nanodomains within the synaptic membrane [46, 47], and these structures are rapidly reorganized in response to synaptic activity [48,49]. Given that tetraspanins are known to associate with each other in membrane domains regulating different trafficking and transduction processes [10], it is tempting to speculate that Tspan6 and Tspan7 are both new components of the PSD scaffold machinery playing an important role in the rearrangement of subsynaptic structures such as AMPA receptors, even within the postsynaptic density. Certainly more experiments, particularly superresolution imaging, may be needed to test this hypothesis.

Further investigation is required to understand the molecular mechanism underlying Tspan6-dependent effect on synaptic transmission and whether this effect is partially responsible for the seizures and intellectual disability described in patients. In addition, in this paper we also showed an effect of Tspan6 deletion in motor learning (S5B Fig) suggesting that Tspan6 is not only playing a role in hippocampal synaptic function but it is also important for other brain regions such as cortex or cerebellum.

## Supporting information

S1 Fig No changes in LTD in the CA3-CA1 synapses from *Tspan6* KO mice. Time course of NMDAR-dependent LTD (1Hz, 15 minutes) in the CA1 neurons from *Tspan6* KO and WT slices. fEPSP slope is normalized to 20 minutes baseline. Insets: representative traces averaged from the baseline (thick lines) or from the last 10 minutes of the recordings (thin lines). *n* = 5 to 7 slices and 5 different mice per group.
(TIF)

S2 Fig *Tspan6* KO hippocampi do not show a compensatory upregulation of Tspan7 protein. Levels of Tspan7 protein were analyzed by western blot in total hippocampal homogenates. n = 5 WT and 5 *Tspan6* KO mice. All lanes belong to the same membrane. Histogram compare mean (±S.E.M) normalized protein levels to loading control (β-actin).
(TIF)

S3 Fig Similar levels of synaptic proteins in *Tspan6* KO hippocampi. **(A)** Purified triton resistant postsynaptic density (PSD) from hippocampal synaptosomes show no difference in GluA1, GluA2 or NR2A receptor subunits. n = 4 WT and 4 *Tspan6* KO mice. **(B)** Phosphorylation state of GluA1 subunit at serine 845 was not changed in *Tpan6* KO hippocampal synaptosomes (n = 7 WT and 7 *Tspan6* KO mice). **(C)** Basal activation of αCamKII by phosphorylation is not increased in *Tspan6* KO synaptosomes. **(D)** Changes in the synaptic levels of mGluR5 were not detected by western blot in hippocampal synaptosomes. **(E)** Levels of glutamate transporter 1 (GLT-1) were analyzed by western blot in total hippocampal homogenates. n = 6 WT and 7 *Tspan6* KO mice. Histograms compare mean (±S.E.M) normalized protein levels to loading control (β-actin or GAPDH).
(TIF)

S4 Fig No differences in anxiety, exploratory or locomotor behavior in 11-months old *Tspan6* KO mice. **(A)**Spontaneous cage activity show no gross alterations in behavioral activity between WT (*n* = 6) and *Tspan6* KO (*n* = 8) mice before the dark (4pm to 8pm) or during the dark phase (from 8pm to 8am, grey block). Histograms compare mean (±S.E.M) number of beam crosses between WT and KO mice. **(B)** In the open field test *Tspan6* KO and control littermates have similar exploratory and locomotor behavior with no changes in distance travelled and number of entries to the center and periphery. Average speed was slightly increased in *Tspan6* KO mice (*p* = 0.07, T-test). *n* = 6 WT, 8 KO mice. **(C)** Elevated plus maze show no difference in the percent of time spent in open arms between *Tspan6* KO (*n* = 19) and control (*n* = 18) mice. Error bars indicate SEM.
(TIF)

S5 Fig *Tspan6* KO mice show no differences in contextual fear conditioning test but impaired motor learning in the rotarod test. **(A)**Contextual fear conditioning in *Tspan6* KO (*n* = 8) and control littermates (*n* = 6). All mice learned to associate the context (increased % of freezing in the context phase) and the tone (increased freezing in the tone phase) with the shocks with no differences between genotypes. **(B)** Rotarod test show impaired motor learning in *Tspan6* KO mice. Latency to fall in the rotating drum is plotted for the different trials (mean ±S.E.M). Test was done after 2 days of training 4 trials a day with 30 and 60 minutes inter-trial interval. *Tspan6* KO mice have a decreased latency to fall during the first trials (F_(3,114)_ = 4.4, p<0.05, Repeated measurements ANOVA). The fact that in the last trial both groups have similar latency values show that *Tspan6* KO do not show any motor deficits and only an effect in learning. *n* = 20 WT and KO mice.
(TIF)
