# Correction: Tetraspanin 6: A novel regulator of hippocampal synaptic transmission and long term plasticity

**DOI:** 10.1371/journal.pone.0178016

**Published:** 2017-07-07

**Authors:** Isabel H. Salas, Zsuzsanna Callaerts-Vegh, Amaia M. Arranz, Francesc X. Guix, Rudi D’Hooge, José A. Esteban, Bart De Strooper, Carlos G. Dotti

The Data Availability statement for this paper is incorrect. The correct statement is: All relevant data are available at the following locations:

Golgi staining: https://doi.org/10.6084/m9.figshare.4730053.v1

Hippocampal primary cultures (spine density): https://doi.org/10.6084/m9.figshare.4730089.v1

qPCR Tspan6 KO mice: https://doi.org/10.6084/m9.figshare.4730038.v1

RNA scope images: https://doi.org/10.6084/m9.figshare.4730026.v1

Electrophysiological recordings: https://doi.org/10.6084/m9.figshare.4730146.v1

Behavioral results: https://doi.org/10.6084/m9.figshare.4730149.v1

Surface GluA1 expression in hippocampal primary neurons: https://doi.org/10.6084/m9.figshare.4730164.v1

Western blot hippocampal synaptosomes: https://doi.org/10.6084/m9.figshare.4730182.v1

Western blot hippocampal homogenates: https://doi.org/10.6084/m9.figshare.4730194.v1
