# Correction: Tetraspanin 6: A novel regulator of hippocampal synaptic transmission and long term plasticity

**DOI:** 10.1371/journal.pone.0187179

**Published:** 2017-10-24

**Authors:** Isabel H. Salas, Zsuzsanna Callaerts-Vegh, Amaia M. Arranz, Francesc X. Guix, Rudi D’Hooge, José A. Esteban, Bart De Strooper, Carlos G. Dotti

Fig 1C and Fig 1D are incorrectly swapped. Please see the corrected Fig 1 here.

**Fig 1:**
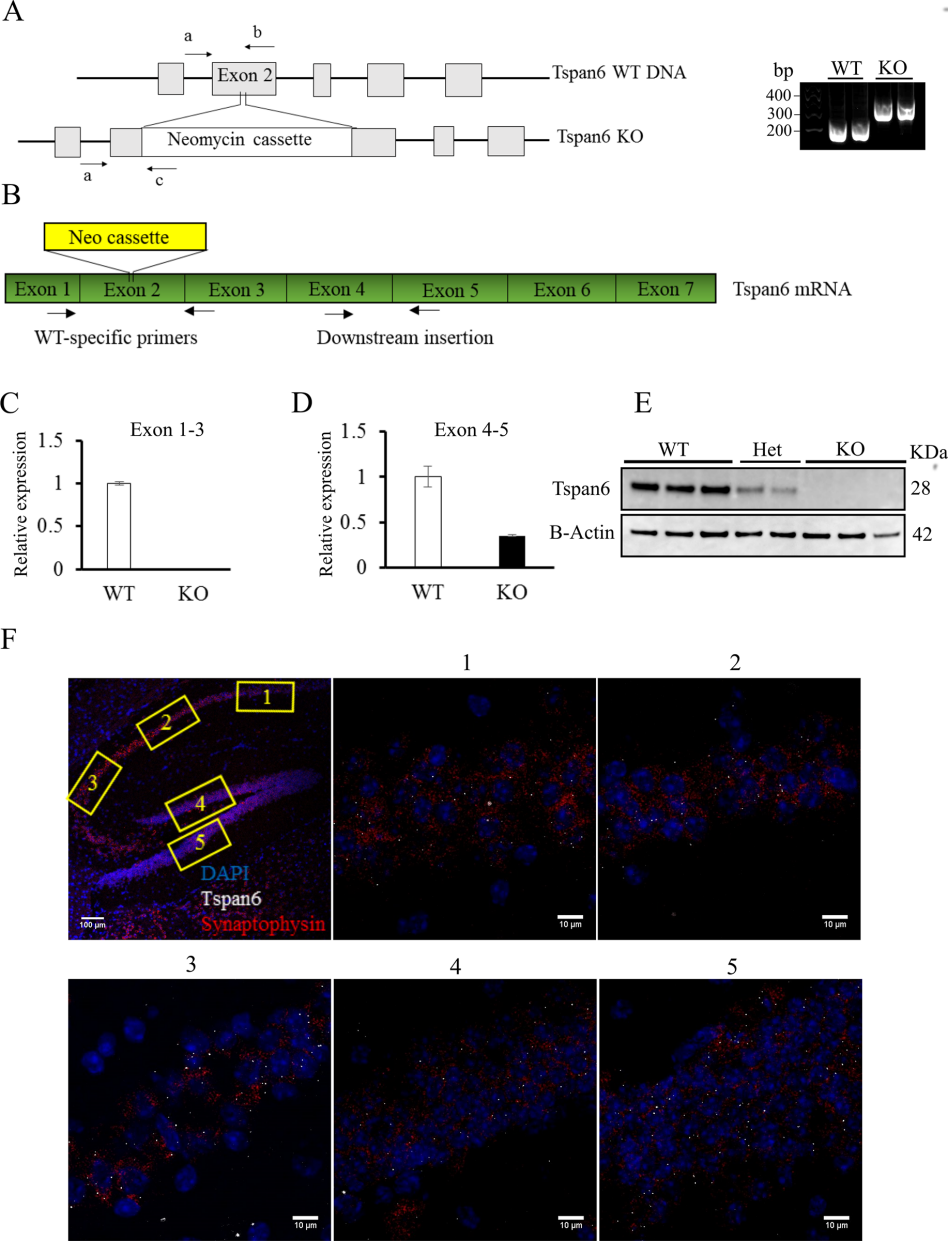
Generation of Tspan6 KO mice and Tspan6 expression in the brain. **(A)** The *Tspan6* KO mouse was generated by insertion of a neomycin cassette in the exon 2 of the *Tspan6* gene. Right panel show a representative agarose gel electrophoresis with the PCR products amplified with specific primers (a, b and c, shown by arrows in the left panel). **(B)** RNA was extracted from *Tspan6* KO and WT animals. Primers were designed between exon 1 and 3 (WT-specific primers), and exon 4 and exon 5 (primers downstream insertion). **(C)** Real time semi-quantitative PCR shows no RNA amplification between exon 1 and 3 in *Tspan6* KO mice due to the insertion of the neomycin cassette. **(D)** RNA amplification downstream the insertion is reduced in *Tspan6* KO mice (0.35± 0.01 mean fold change compared to WT) suggesting RNA degradation. Histogram shows mean (±S.E.M) fold changes normalized against WT expression, using either WT-specific primers (C) or primers downstream the insertion (D). Two housekeeping genes (Actin and GAPDH) were used for the normalization of the expression. **(E)** Neuronal lysates from cortical primary cultures from *Tspan6* WT, heterozygous and KO mice show the absence of Tspan6 protein in the KO condition. **(F)** RNA scope shows expression of Tspan6 RNA in the pyramidal layer of the hippocampus and granule cells from the dentate gyrus. First panel is a general view of the hippocampus (scale bar = 100μm). Panels 1 to 5 show box section in higher magnification (scale bar = 10μm).White dots are Tspan6 RNA molecules, synaptophysin RNA is stained in red and DAPI in blue.
