# Supplementary figures and images for: Tetraspanin 6: A novel regulator of hippocampal synaptic transmission and long term plasticity
Source: PLoS One. 2017 Feb 16;12(2):e0171968. doi: 10.1371/journal.pone.0171968 (PMC5312877; doi:10.1371/journal.pone.0171968)

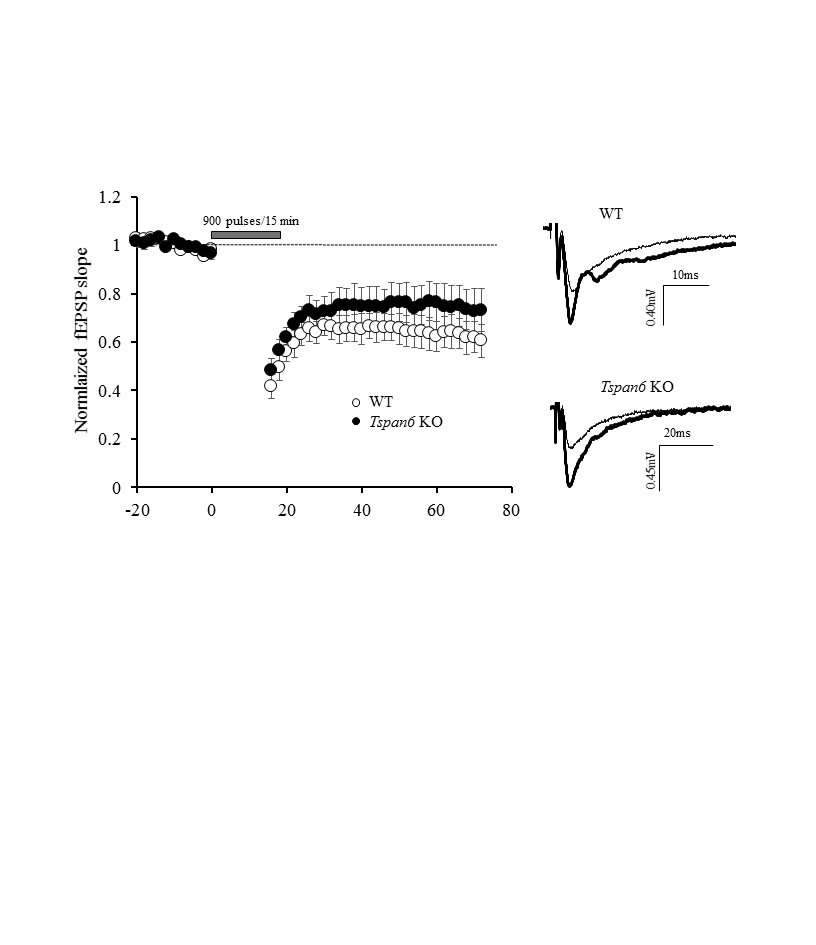

Supplement: S1 Fig — Time course of NMDAR-dependent LTD (1Hz, 15 minutes) in the CA1 neurons from Tspan6 KO and WT slices. fEPSP slope is normalized to 20 minutes baseline. Insets: representative traces averaged from the baseline (thick lines) or from the last 10 minutes of the recordings (thin lines). n = 5 to 7 slices and 5 different mice per group. (TIF) [file pone.0171968.s001.TIF]

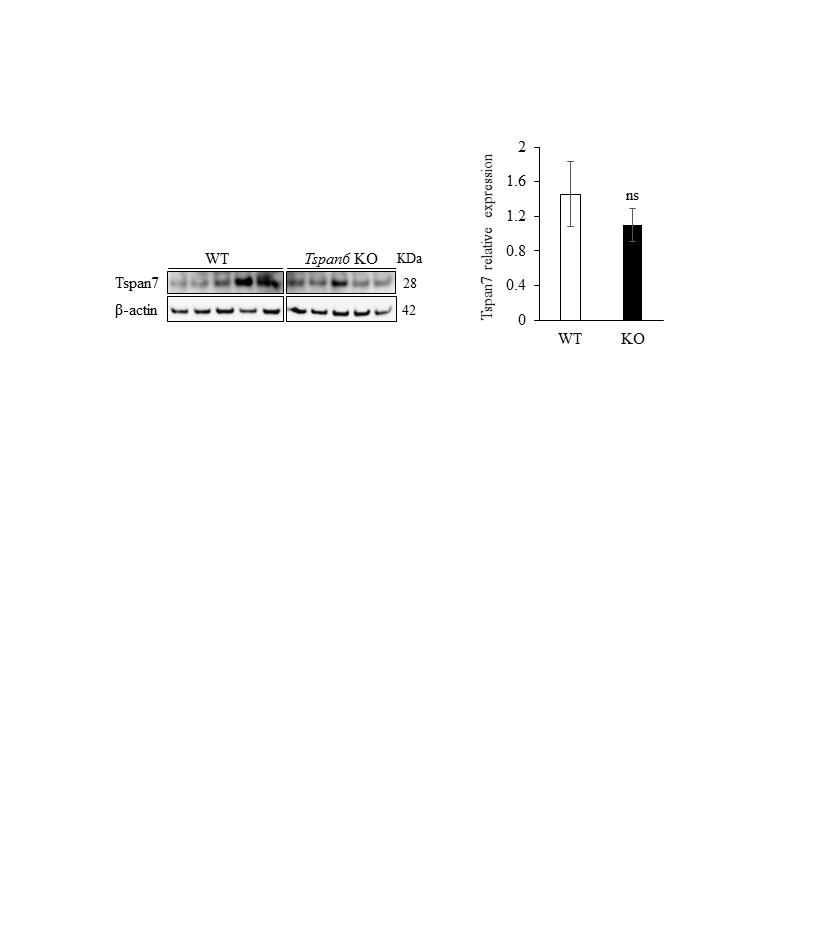

Supplement: S2 Fig — Levels of Tspan7 protein were analyzed by western blot in total hippocampal homogenates. n = 5 WT and 5 Tspan6 KO mice. All lanes belong to the same membrane. Histogram compare mean (±S.E.M) normalized protein levels to loading control (β-actin). (TIF) [file pone.0171968.s002.TIF]

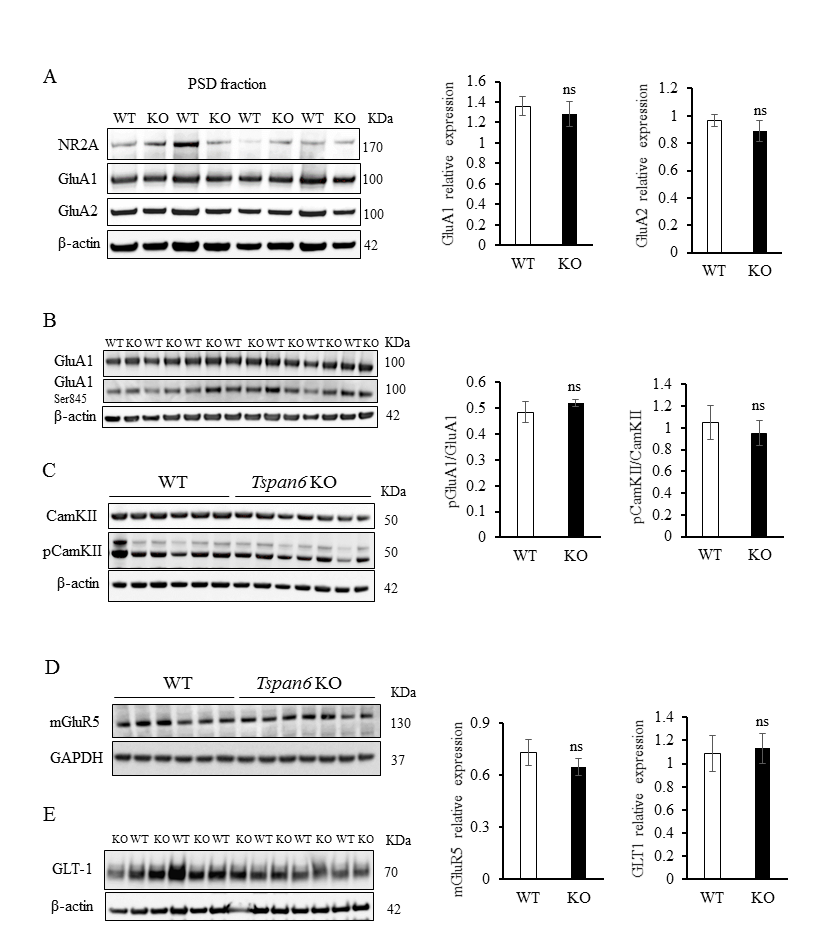

Supplement: S3 Fig — (A) Purified triton resistant postsynaptic density (PSD) from hippocampal synaptosomes show no difference in GluA1, GluA2 or NR2A receptor subunits. n = 4 WT and 4 Tspan6 KO mice. (B) Phosphorylation state of GluA1 subunit at serine 845 was not changed in Tpan6 KO hippocampal synaptosomes (n = 7 WT and 7 Tspan6 KO mice). (C) Basal activation of αCamKII by phosphorylation is not increased in Tspan6 KO synaptosomes. (D) Changes in the synaptic levels of mGluR5 were not detected by western blot in hippocampal synaptosomes. (E) Levels of glutamate transporter 1 (GLT-1) were analyzed by western blot in total hippocampal homogenates. n = 6 WT and 7 Tspan6 KO mice. Histograms compare mean (±S.E.M) normalized protein levels to loading control (β-actin or GAPDH). (TIF) [file pone.0171968.s003.TIF]

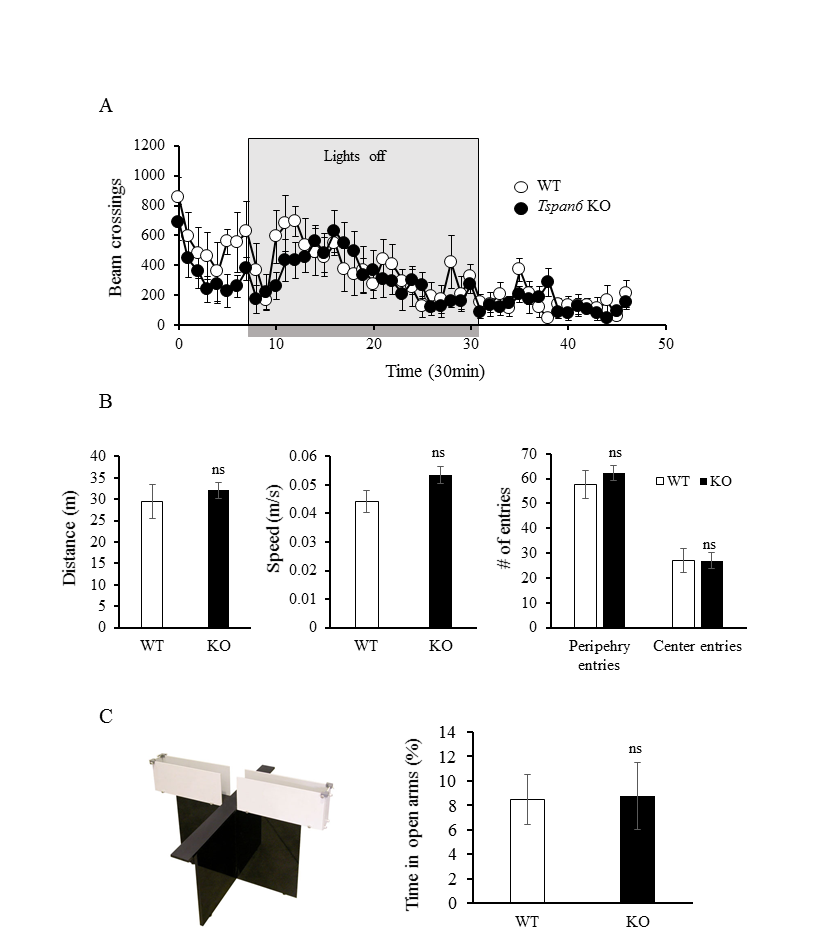

Supplement: S4 Fig — (A)Spontaneous cage activity show no gross alterations in behavioral activity between WT (n = 6) and Tspan6 KO (n = 8) mice before the dark (4pm to 8pm) or during the dark phase (from 8pm to 8am, grey block). Histograms compare mean (±S.E.M) number of beam crosses between WT and KO mice. (B) In the open field test Tspan6 KO and control littermates have similar exploratory and locomotor behavior with no changes in distance travelled and number of entries to the center and periphery. Average speed was slightly increased in Tspan6 KO mice (p = 0.07, T-test). n = 6 WT, 8 KO mice. (C) Elevated plus maze show no difference in the percent of time spent in open arms between Tspan6 KO (n = 19) and control (n = 18) mice. Error bars indicate SEM. (TIF) [file pone.0171968.s004.TIF]

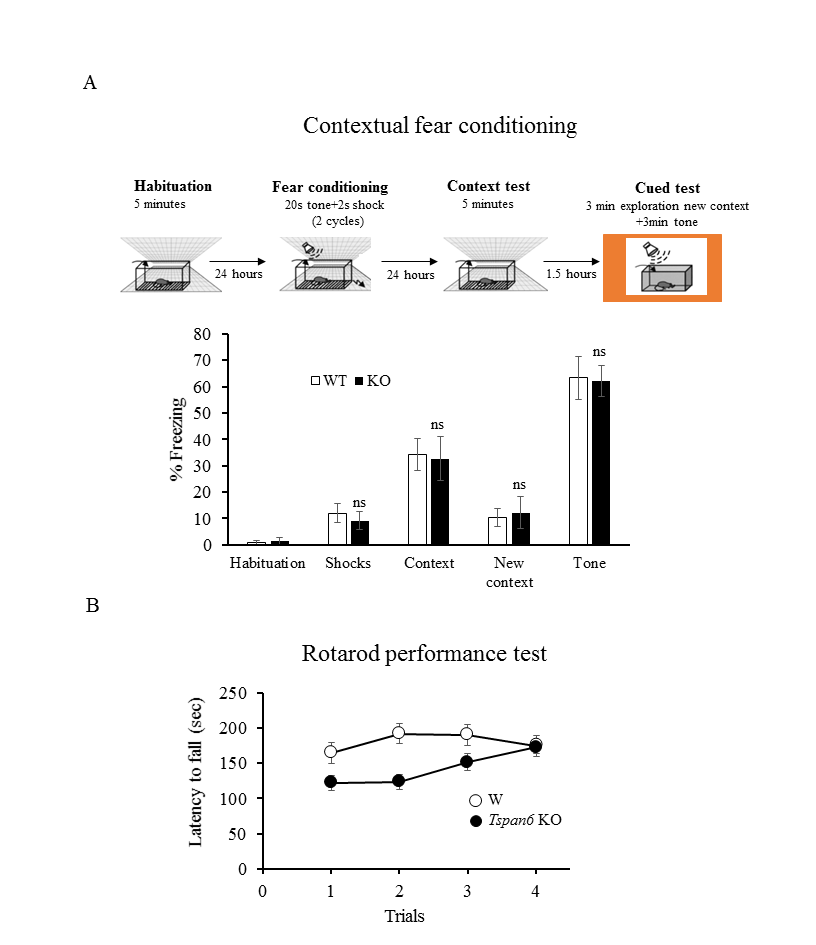

Supplement: S5 Fig — (A)Contextual fear conditioning in Tspan6 KO (n = 8) and control littermates (n = 6). All mice learned to associate the context (increased % of freezing in the context phase) and the tone (increased freezing in the tone phase) with the shocks with no differences between genotypes. (B) Rotarod test show impaired motor learning in Tspan6 KO mice. Latency to fall in the rotating drum is plotted for the different trials (mean ±S.E.M). Test was done after 2 days of training 4 trials a day with 30 and 60 minutes inter-trial interval. Tspan6 KO mice have a decreased latency to fall during the first trials (F(3,114) = 4.4, p<0.05, Repeated measurements ANOVA). The fact that in the last trial both groups have similar latency values show that Tspan6 KO do not show any motor deficits and only an effect in learning. n = 20 WT and KO mice. (TIF) [file pone.0171968.s005.TIF]
